# Evolving Defect Chemistry of (Pu,Am)O_2±*x*_

**DOI:** 10.1021/acs.jpcc.1c03274

**Published:** 2021-07-07

**Authors:** William D. Neilson, Helen Steele, Samuel T. Murphy

**Affiliations:** † Engineering Department, Lancaster University, Bailrigg, Lancaster LA1 4YW, U.K.; ‡ Sellafield Ltd., Sellafield, Cumbria CA20 1PG, U.K.

## Abstract

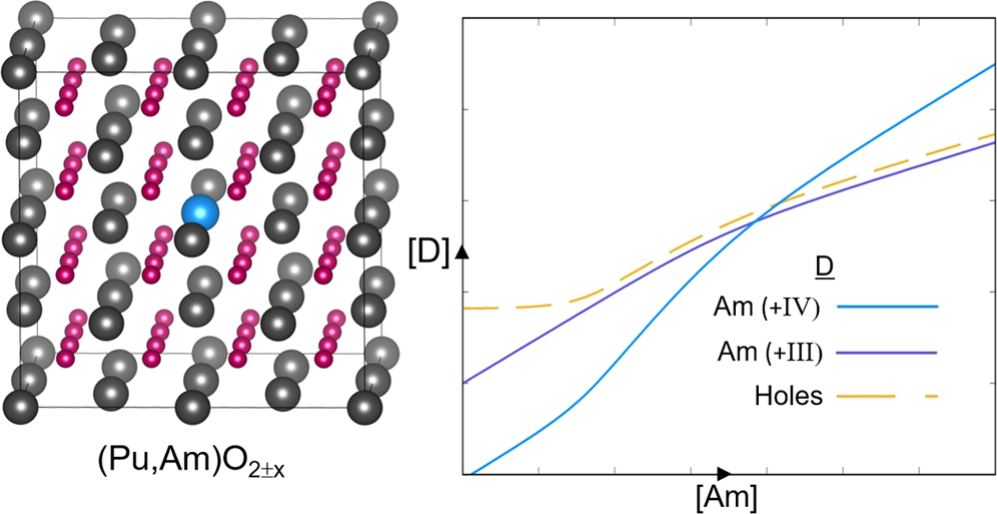

The β decay of ^241^Pu to ^241^Am results in a significant ingrowth of Am during the interim storage of PuO_2_. Consequently, the safe storage of the large stockpiles of separated Pu requires an understanding of how this ingrowth affects the chemistry of PuO_2_. This work combines density functional theory (DFT) defect energies and empirical potential calculations of vibrational entropies to create a point defect model to predict how the defect chemistry of PuO_2_ evolves due to the incorporation of Am. The model predicts that Am occupies Pu sites in (Pu,Am)O_2±*x*_ in either the +III or +IV oxidation state. High temperatures, low oxygen-to-metal (O/M) ratios, or low Am concentrations favor Am in the +III oxidation state. Am (+III) exists in (Pu,Am)O_2±*x*_ as the negatively charged (Am_Pu_^1–^) defect, requiring charge compensation from holes in the valence band, thereby increasing the conductivity of the material compared to Am-free PuO_2_. Oxygen vacancies take over as the charge compensation mechanism at low O/M ratios. In (Pu,Am)O_2±*x*_, hypo- and (negligible) hyperstoichiometry is found to be provided by the doubly charged oxygen vacancy (V_O_^2+^) and singly charged oxygen interstitial (O_i_^1–^), respectively.

## Introduction

The management of the large stockpiles of Pu, separated from spent nuclear fuel or nuclear weapons programs, pose a series of technical challenges associated with its potential storage, disposal, and reuse. In particular, oxidation of the material during interim storage and the formation of hyperstoichiometric PuO_2+*x*_ may initiate chemical reactions that cause potential pressurization of PuO_2_ storage canisters.^1^ Previous theoretical investigations of the defect chemistry of PuO_2_ suggest that pure PuO_2_ is very reluctant to form hyperstoichiometric PuO_2+*x*_.^2^ However, under storage conditions, aged PuO_2_ contains significant ingrowth of Am produced by ^241^Pu decaying into ^241^Am. Am builds up relatively quickly due to the short half-life of ^241^Pu (14.4 years), with Am concentrations peaking after approximately 70 years, at which point, it too begins to decay faster than it is produced.^3^ Incorporation of Am is predicted to alter the defect chemistry of PuO_2_; in a density functional theory (DFT) investigation on Pu–Am mixed oxide surfaces, Chen et al.^4^ report that the presence of Am promotes the formation of O vacancies that increase the favorability of molecular adsorption of water on PuO_2_ surfaces while reducing the favorability of dissociative water adsorption. The consequence of this could be an increased likelihood of chemical reactions including the aforementioned pressurization.^4^

Pu–Am mixed oxides have also been investigated as a fuel candidate in the design of the fourth generation (GEN-IV) of nuclear reactors. The oxygen-to-metal O/M ratio is an important parameter of the fuel and influences multiple thermophysical properties, including the oxygen potential. Osaka et al.^5^ experimentally determined the oxygen potential of (Pu_0.91_Am_0.09_)O_2–*x*_ as a function of the O/M ratio, as well as measuring the deviation *x* from stoichiometry as a function of oxygen partial pressure. Matsumoto et al.^6^ experimentally studied the oxygen potential of (Pu_0.928_Am_0.072_)O_2–*x*_ at high temperatures as a function of the O/M ratio and constructed point defect equations to describe the deviation *x* from stoichiometry. A doubly charged vacancy is predicted as the source of all hypostoichiometry.^6^ Using X-ray absorption spectroscopy, Belin et al.^7^ were able to quantitatively determine Pu and Am valences in the reduction process of (Pu,Am)O_2–*x*_, validating an earlier prediction made by Osaka et al.^5^ that all Am (+IV) will reduce to Am (+III) prior to any reduction in Pu (+IV) occurring.

Globally, significant amounts of Pu exist in the environment, a proportion of which is the form of PuO_2_.^8^ The subsurface mobility of the material is very complex and is likely impacted by the accumulation of Am, which has a different environmental mobility.^8,9^^238^PuO_2_ is also the most commonly used isotope in radioisotope thermoelectric generators and heating units for space applications.^10^ A better understanding of how PuO_2_ continues to evolve and accommodates Am growth is, therefore, of widespread interest. In particular, the oxidation state that Am adopts in PuO_2_ is of importance as it will alter, to some degree, the materials surface reactivity, thermophysical properties, and environmental mobility. Here, we construct a point defect model from DFT to investigate the mode of Am incorporation within PuO_2_ and the impact its presence has on the defect chemistry. Using the model, we are able to predict how the stoichiometry in (Pu_1–*y*_Am_*y*_)O_2±*x*_ changes in response to Am ingrowth under a range of environmental conditions (oxygen partial pressure and temperature).

As with other actinide oxides, the application of DFT to study plutonium oxides must be approached with care. Use of conventional semilocal functionals results in the self-interaction error that causes PuO_2_ to be described as conducting as opposed to its correct classification as a charge-transfer insulator.^11^ This is caused by an over delocalization of the 5f electrons.^11^ Multiple approaches exist to overcome this shortcoming, two of these are the use of hybrid functionals and the DFT + *U* method. Using hybrid functionals, good reproduction of the experimental properties of PuO_2_ has been achieved.^12,13^ Hybrid functionals blend a portion of the Hartree–Fock (HF) exchange into a part of a density functional; they are known to offer significantly improved descriptions of band gaps, especially in small- to medium-gap systems (<5 eV).^14^ The DFT + *U* method has been applied more extensively due to its lower computational cost in comparison to hybrid functionals. DFT + *U* models require the selection of *U* and *J* as input parameters and are usually obtained by fitting to the structural and electronic properties of PuO_2_.^12,15−19^ The determination of appropriate values for *U* and *J* is made difficult by the variation in experimental properties reported in the literature, in particular the value of the electronic band gap. McNeilly et al.^20^ reported a value for the band gap of 1.8 eV, while more recently, Mark McCleskey et al.^21^ reported a higher value of 2.8 eV. Consequently, a wide variety of *U* and *J* values have been used in previous DFT simulations of PuO_2_.

## Methodology

### Computational Procedure

DFT simulations were performed using the Vienna *Ab initio* Simulation Package (VASP)^22−25^ employing the projector augmented wave (PAW)^26,27^ method implemented with the frozen-core approximation. The plutonium and americium 6s, 6p, 5f, 6d, and 7s, and oxygen 1s, 2s, and 2p electrons are treated as valence. Following convergence testing, the cutoff energy for the plane-wave basis set was selected to be 500 eV and a 2 × 2 × 2 Monkhorst–Pack *k*-point mesh^28^ was used for the 96-atom PuO_2_ supercells. The noncollinear relativistic computational study of the PuO_2_ magnetic structure by Pegg et al.^12^ finds a longitudinal 3k antiferromagnetic (AFM) magnetic ground state for PuO_2_, which was adopted in this study. Spin–orbit interaction (SOI)^29^ is considered as not including it resulted in a different magnetic ground state being obtained.^12^

For the calculation of defect energies in PuO_2_ supercells, we apply the DFT + *U* method using the Liechtenstein approach.^30^ DFT + *U* calculations are performed with the generalized gradient approximation (GGA) formulation of Perdew–Burke–Ernzerhof functional revised for solids (PBEsol + *U*).^31,32^ The energy threshold for electronic convergence is set as 1 × 10^–6^ eV with structural convergence deemed complete when the forces on all atoms did not exceed 2 × 10^–2^ eV Å^–1^. The *U* parameter of the PBEsol + *U* approximation was set at 7.0 eV, selected to reproduce the band gap obtained from the hybrid Heyd–Scuseria–Ernzerhof (HSE06) functional.^33−36^ The HSE06 functional achieves good reproduction of experimental structural properties of PuO_2_ and predicts an electronic band gap of 3.04 eV.^12^ The *J* parameter was fixed at a value of 0.0 eV throughout this study, as any introduction of *J* was shown to detrimentally affect the reproduction of the band gap for PuO_2_.^15^ The decision and justification for the selection of our *U* and *J* parameters are discussed in detail in our previous work, which also reports the equilibrium properties the HSE06 and PBEsol + *U* (*U* = 7.0 eV) functionals attain simulating PuO_2_.^2^ In summary, it was chosen to reproduce the HSE06 band gap to set *U* as the experimental data shows a large variation, and this functional has been proven to replicate experimental band gaps.^37^ In the Supporting Information, we present a comparison of the projected densities of states (DOS) obtained using the PBEsol + *U* and HSE06 functionals as well as evidence that while the choice of *U* impacts the DOS, the impact to the DFT formation energy of a defect is minimal.

The defects considered in this study are presented in Table 1. For the defects studied here, only one unique site exists in the supercell, due to the symmetry of the PuO_2_*Fm*3̅*m* lattice. By adding or removing electrons from the supercell, variation in the charges of the defects can be studied. Defect-containing supercells were relaxed under constant volume, using the lattice constants obtained from defect-free simulations.

**Table 1:** Defects Studied in This Investigation, Represented with Kröger–Vink Notation,^38^ Modified to Display Charge as an Integer Value (No Charge Indicated by ×)

defect		charge states
oxygen	interstitials	O_i_^×^, O_i_^1–^, O_i_^2–^
	vacancies	V_O_^×^, V_O_^1+^, V_O_^2+^
plutonium	interstitials	Pu_i_^×^, Pu_i_^1+^, Pu_i_^2+^, Pu_i_^3+^, Pu_i_^4+^
	vacancies	V_Pu_^×^, V_Pu_^1–^, V_Pu_^2–^, V_Pu_^3–^, V_Pu_^4–^
americium	interstitials	Am_i_^×^, Am_i_^1+^, Am_i_^2+^, Am_i_^3+^, Am_i_^4+^
	Pu substitutions	Am_Pu_^4–^, Am_Pu_^3–^, Am_Pu_^2–^, Am_Pu_^1–^, Am_Pu_^×^, Am_Pu_^1+^, Am_Pu_^2+^, Am_Pu_^3+^, Am_Pu_^4+^, Am_Pu_^5+^
	O substitutions	Am_O_^×^, Am_O_^1+^, Am_O_^2+^, Am_O_^3+^, Am_O_^4+^, Am_O_^5+^, Am_O_^6+^

To provide reference for the Am oxidation state and to assess the thermodynamical stability of (Pu,Am)O_2±*x*_, AmO_2_ and Am_2_O_3_ were simulated with DFT. The PBEsol + *U* functional is used with *U* set to 4 eV, SOI considered, and a 5 × 5 × 5 *k*-point mesh applied. For AmO_2_, transverse 3k AFM order is applied,^15^ while we simulate A-type Am_2_O_3_ with longitudinal 1k AFM order.^39^ The bulk properties produced with these simulation parameters are reasonable compared to experiment (see the Supporting Information).

### Vibrational Entropies

Following Cooper et al.^40^ and Soulié et al.,^41^ when calculating the defects formation energy, we consider the difference in vibrational entropy between defective and perfect supercells (Δ*S*_vib_). Vibrational entropies are obtained using empirical potentials as the required phonon calculations become very expensive in defect-containing supercells. The General Utility Lattice Program (GULP)^42^ together with the Cooper, Rushton, and Grimes (CRG)^43,44^ potential is adopted. The CRG potential is a many-body potential model used to describe actinide oxide systems that achieves good reproduction of thermodynamic and mechanical properties. Previous work demonstrated that the phonon DOS for PuO_2_ produced using the CRG potential compares reasonably well to the experimental data of Manley et al.^2,45^ The calculation of vibrational entropies, *S*_vib_, closely follows the approach described in refs (40, 46, 47), where using eq 1, defect entropies are calculated from the normal vibrational frequencies, *v*_n_, which are themselves calculated by diagonalizing the dynamical matrix of the system
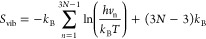
1In this formula, *h* is Planck’s constant, *N* is the number of atoms in the crystal, *T* is the temperature, and *k*_B_ is the Boltzmann constant. In this study, the system to calculate vibrational entropies is a 4 × 4 × 4 expansion of the PuO_2_ unit cell. Defective supercells are created by adding or removing atoms and are relaxed under constant volume. Defect vibrational entropies are found by calculating the difference in vibrational entropies between the defective and perfect supercell (Δ*S*_vib_). The Δ*S*_vib_ values calculated for the Am extrinsic defects are presented in Table 2, and the Δ*S*_vib_ values for the intrinsic defects remain the same as in ref (2). As the charges assigned to the ions in an empirical simulation are fixed, the same value of Δ*S*_vib_ is given to all charge states of a given defect.

**Table 2:** Difference in Vibrational Entropy of PuO_2_ due to the Addition of Am Extrinsic Defects, Calculated Using the CRG Potential

	defect entropy (Δ*S*_vib_/*k*_B_)		
*T* (K)	Am_i_	Am_Pu_	Am_O_
400	6.835	–0.081	5.268
600	7.322	–0.081	4.711
800	7.810	–0.081	4.479
1000	8.332	–0.081	4.363
1200	8.796	–0.081	4.305
1400	9.307	–0.081	4.259
1600	9.667	–0.070	4.236
1800	9.922	–0.070	4.224
2000	10.305	–0.081	4.201

### Charge Corrections

As discussed extensively in ref (48), the introduction of charged defects into the small simulation supercells accessible using DFT introduces a number of finite size effects. These include Coulombic interactions between the defect and its periodic image as well as with the neutralizing background charge. The result is that defect formation energies exhibit a strong dependence on the size of the supercell used, and this must be corrected for when calculating a defect’s formation energy. Several correction methods exist; it was previously found that the scheme developed by Kumagai and Oba^49^ was very successful at accounting for finite size effects exhibited in PuO_2_, and therefore it is applied in this work.^2^ The scheme of Kumagai and Oba is an extension of that developed by Freysoldt et al.^50^ and uses the atomic site electronic potentials of supercells with (*V*_defect,q_) and without (*V*_bulk_) defects to calculate the correction. The energy correction, *E*_corr_, for a defect with charge *q* is summarized following ref (49) as
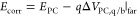
2
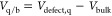
3
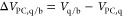
4where Δ*V*_PC,q/b_ is the potential difference between the defect induced potential *V*_q/b_ and the point charge (PC) potential *V*_PC,q_.^50^ Δ*V*_PC,q/b_|_far_ is Δ*V*_PC,q/b_ at a position far from the defect site but still within the supercell. For a cubic system, such as PuO_2_, the PC correction energy (*E*_PC_) can be expressed as^51^
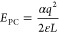
5where α = 2.837 is the lattice-type-dependent Madelung constant, *L* is the supercell lattice constant, and ε is the static dielectric constant. The static dielectric constant of PuO_2_ was calculated using density functional perturbation theory (DFPT)^52,53^ as implemented in VASP giving a value of 19.66.^2^

### Defect Formalism

The defect formation energy, Δ*G*_f_^i^, for a defect, *i*, is given by eq 6
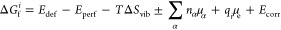
6where *E*_def_ and *E*_perf_ are the DFT total energies of the defective and perfect supercells, *n*_α_ is the number of atoms of species, α, added to or removed from the system to make defect *i*, μ_α_ is the chemical potential of species α, and μ_e_ is the electron chemical potential. Using Boltzmann statistics, the concentration of defect *i*, *c*_*i*_, can be calculated using the formation energy of defect *i* and its multiplicity, *m_i_*
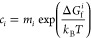
7The electron chemical potential, μ_e_ = *E*_VBM_ + ε_F_, is expressed as the sum of the energy of the valence band maximum (VBM), *E*_VBM_, and the electron chemical potential above the VBM, ε_F_. As overall charge neutrality of the system must be maintained, the concentrations of ionic and electronic defects must be such that at any given temperature and oxygen partial pressure, the following criterion is met^54^
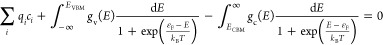
8The first term is the sum of the charges of the point defects. The second and third terms are determined by applying Fermi–Dirac statistics to the electronic DOS to obtain the concentrations of electrons (e^–^) in the conduction band and concentration of holes (p^+^) in the valence band, respectively. Within these two integrals are *g*_v_(*E*) and *g*_c_(*E*), the density of electronic states in the valence band and conduction band per formula unit of PuO_2_, respectively. For calculation of the electron population, *E*_CBM_ is the energy of the conduction band minimum. The Defect Analysis Package^55^ employs linear bisection to find the value of ε_F_ that ensures charge neutrality for any given oxygen partial pressure and temperature. This enables plotting of the concentration of a defect as a function of the oxygen partial pressure or temperature. Additionally, the calculated defect concentrations are used to calculate the deviation in stoichiometry, *x* in (Pu,Am)O_2±*x*_. Using the concentration of a defect summed over all charge states, *w*, *y*, and *z* in Pu_1+*w*_Am_*y*_O_2+*z*_ are given by
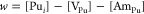
9
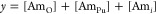
10
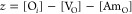
11*x* in (Pu_1–*y*_Am_*y*_)O_2+*x*_ or −*x* in (Pu_1–*y*_Am_*y*_)O_2–*x*_ can be defined as
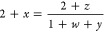
12
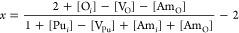
13

### Chemical Potentials

The chemical potentials of plutonium, μ_Pu_(*P*_O_2__,*T*), and oxygen, μ_O_2__(*P*_O_2__,*T*) are defined using the chemical potential of solid PuO_2_ (μ_PuO_2(s)__)
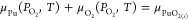
14For a solid μ(*P*_O_2__°,*T*°) ≈ μ(0,0), therefore, the temperature and pressure dependencies have been dropped. Under equilibrium conditions, the chemical potential of Pu cannot exceed that of solid Pu, otherwise a Pu precipitate would form. This upper bound is the Gibbs free energy of Pu in its natural state. It can therefore be said that under Pu-rich conditions
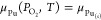
15To find μ_Pu_(s)__, we simulate the α phase of Pu with DFT using PBEsol + *U*. We use the recommendation of the review by Söderlind et al.^56^ to use small *U* and *J* values, setting *U* and *J* parameters at 2.2 and 0.58 eV, respectively. The atomic volume obtained with these values (18.27 Å^3^) matched closely the atomic volume obtained by Söderlind et al.^56^ when using PBE + *U*. To determine the chemical potential of oxygen, the approach of Finnis et al.^57^ is adopted. This method uses the known experimental formation energy of the oxide (Δ*H*_f_^PuO_2_^ (*P*_O_2__°,*T*°) = −10.94 eV^58^) to obtain the chemical potential of oxygen at standard temperature and pressure, i.e.,
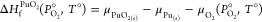
16Unlike the solid species in eq 16, the temperature and pressure dependence of the oxygen chemical potential cannot be neglected and is extrapolated from μ_O_2__(*P*_O_2__°,*T*°) using formulas derived by Johnston et al.^59^
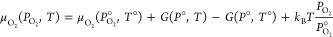
17where the temperature-dependent Gibbs free energy per mole is fitted to a polynomial derived from experimental data (coefficients listed in Table 3)^59^
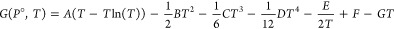
18While the chemical potential of americium (μ_Am_(*P*_O_2__,*T*)) can be determined from DFT, here, the chemical potential is fitted to reproduce the desired concentration of Am, allowing for a comparison with experiment. μ_Am_(*P*_O_2__,*T*) is determined using a linear bisection in the Defect Analysis Package.^55^

**Table 3:** Coefficients for Gibbs Free Energy Expression in Eq 18(59)

*A*	29.659 × 10^–3^ kJ mol^–1^ K^–1^
*B*	6.137261 × 10^–6^ kJ mol^–1^ K^–2^
*C*	–1.186521 × 10^–9^ kJ mol^–1^ K^–3^
*D*	0.095780 × 10^–12^ kJ mol^–1^ K^–4^
*E*	–0.219663 × 10^3^ kJ mol^–1^ K
*F*	–9.861391 kJ mol^–1^
*G*	237.948 × 10^–3^ kJ mol^–1^ K^–1^

## Results and Discussion

**Figure 1:**
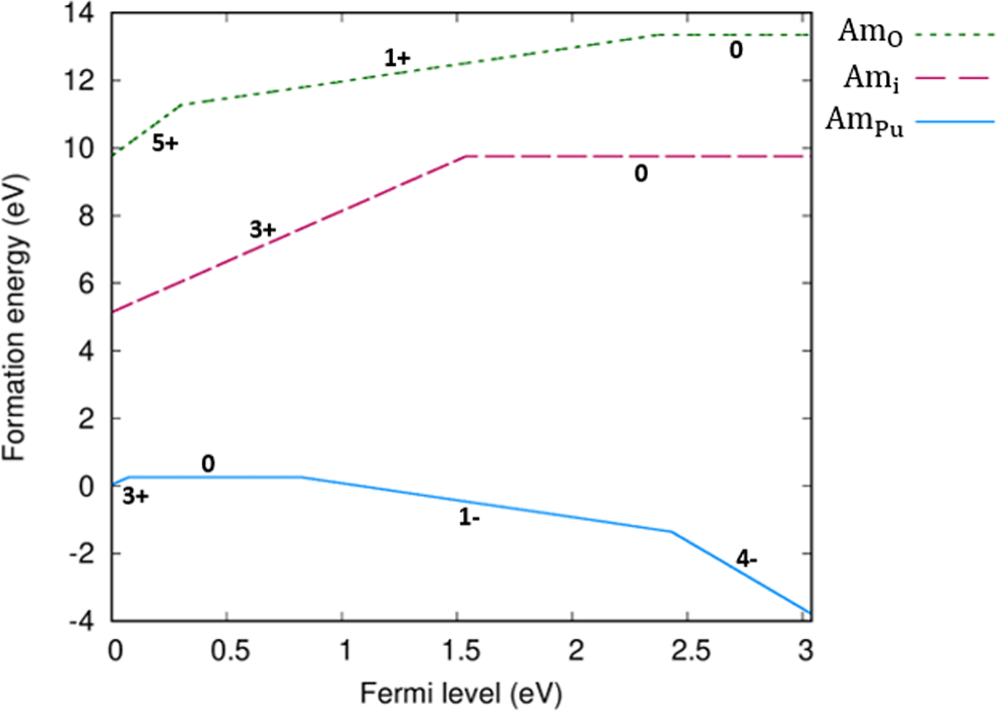
Defect formation energies for Am_Pu_, Am_O_, and Am_i_ defects in (Pu_1–*y*_Am_*y*_)O_2±*x*_ (*y* = 0.001) as a function of Fermi energy. Calculated at 1000 K and an oxygen partial pressure of 0.10 atm. Only the charge state with the lowest formation energy for a given Fermi level is shown for each defect, represented with numeric label.

**Figure 2:**
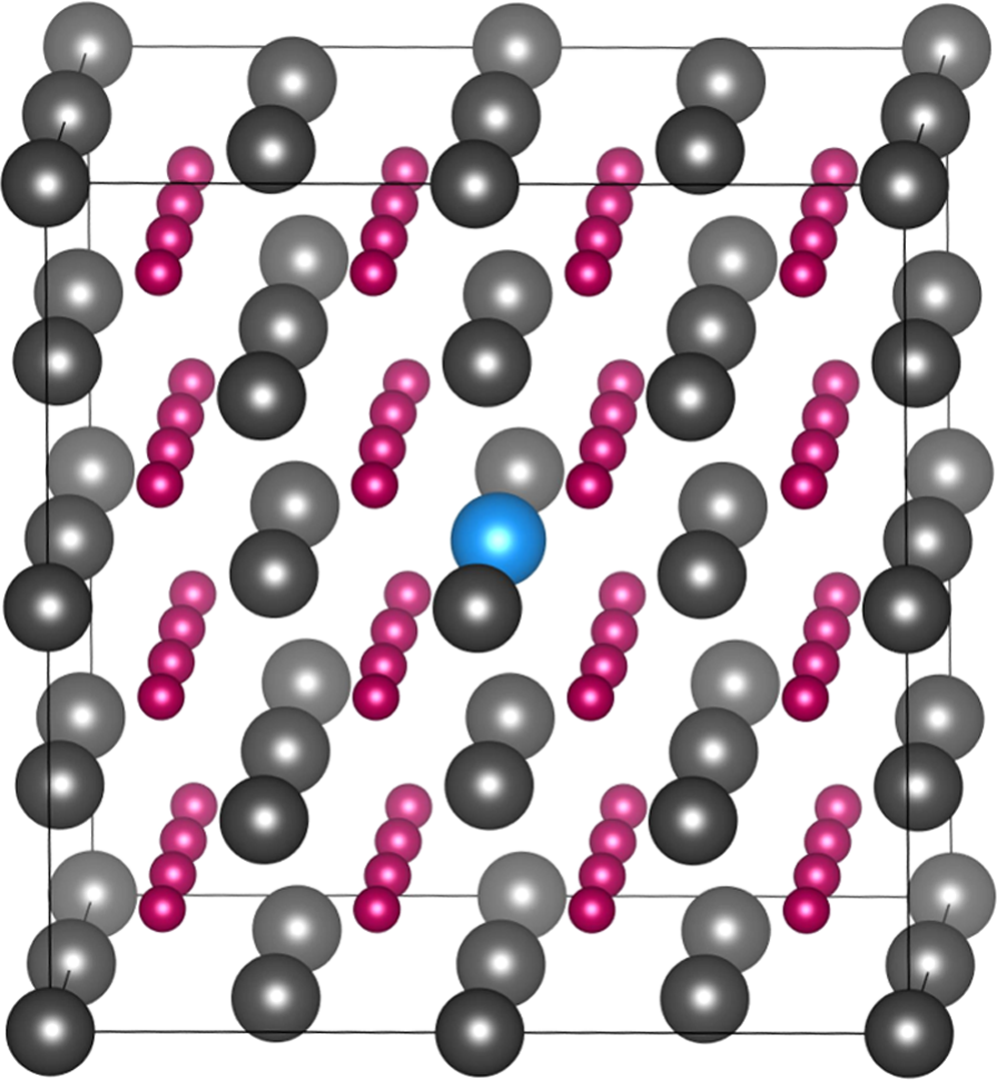
Final relaxed structure for the Am_Pu_^1–^ defect in PuO_2_. Plutonium, americium, and oxygen are represented with gray, blue, and red spheres, respectively.

The formation energies of the Am-based extrinsic defects are plotted as a function of the Fermi level at 1000 K and an oxygen partial pressure of 0.10 atm in Figure 1. Figure 1 displays the charge state of each defect that corresponds to the lowest formation energy at a given position in the band gap. A similar plot for the intrinsic defects is given in previous work.^2^ It is clear from Figure 1 that the Am_Pu_ defects have significantly lower formation energies than Am_O_ and Am_i_ defects across the whole band gap. This result is found to be consistent at both high and low oxygen partial pressures and Am concentrations. This shows that in (Pu,Am)O_2_, Am is preferentially accommodated as a substitutional defect on the Pu site. The most energetically stable charge state of Am_Pu_ is seen to vary across the band gap, with four states in total dominant at one time. However, it is the Am_Pu_^1–^ and Am_Pu_^×^ charge states that dominate across the majority of the band gap, suggesting that it is these two defects that accommodate americium under most equilibrium conditions. By studying the electron occupation of the Am atom in the simulated defect-containing supercells, it was possible to infer an oxidation state for americium of +III and +IV in Am_Pu_^1–^ and Am_Pu_^×^, respectively. The results of a Bader charge analysis^60^ (Table 4) helps us to confirm this result, using AmO_2_ and Am_2_O_3_ as reference oxides for the Am (IV) and Am (III) charge states, respectively. Pu remains in the +IV oxidation state, regardless of defect. Am_Pu_ defects do not cause significant distortion of the PuO_2_ lattice; only a small distortion of the eight nearest oxygen atoms is observed, as shown in Figure 2. In cells containing the Am_Pu_^1–^ defect, the O–Am bond length is just 0.05 Å higher than the O–Pu bond length of 2.34 Å in Am-free PuO_2_; this increase is lower in cells containing the Am_Pu_^×^ defect. This is explained and supported by the reported crystallography: Am (+IV) and Am (+III) with eightfold coordination have ionic radii of 0.95 and 1.09 Å, respectively.^61^ Pu (+IV) with eightfold coordination has an ionic radius of 0.96 Å.^61^

**Table 4:** Bader Charge of Am in Am_Pu_ Defects and Am Oxides

Am_Pu_^×^	2.44
Am_Pu_^1–^	2.02
AmO_2_ (Am (IV))	2.38
Am_2_O_3_ (Am (III))	1.96

**Figure 3:**
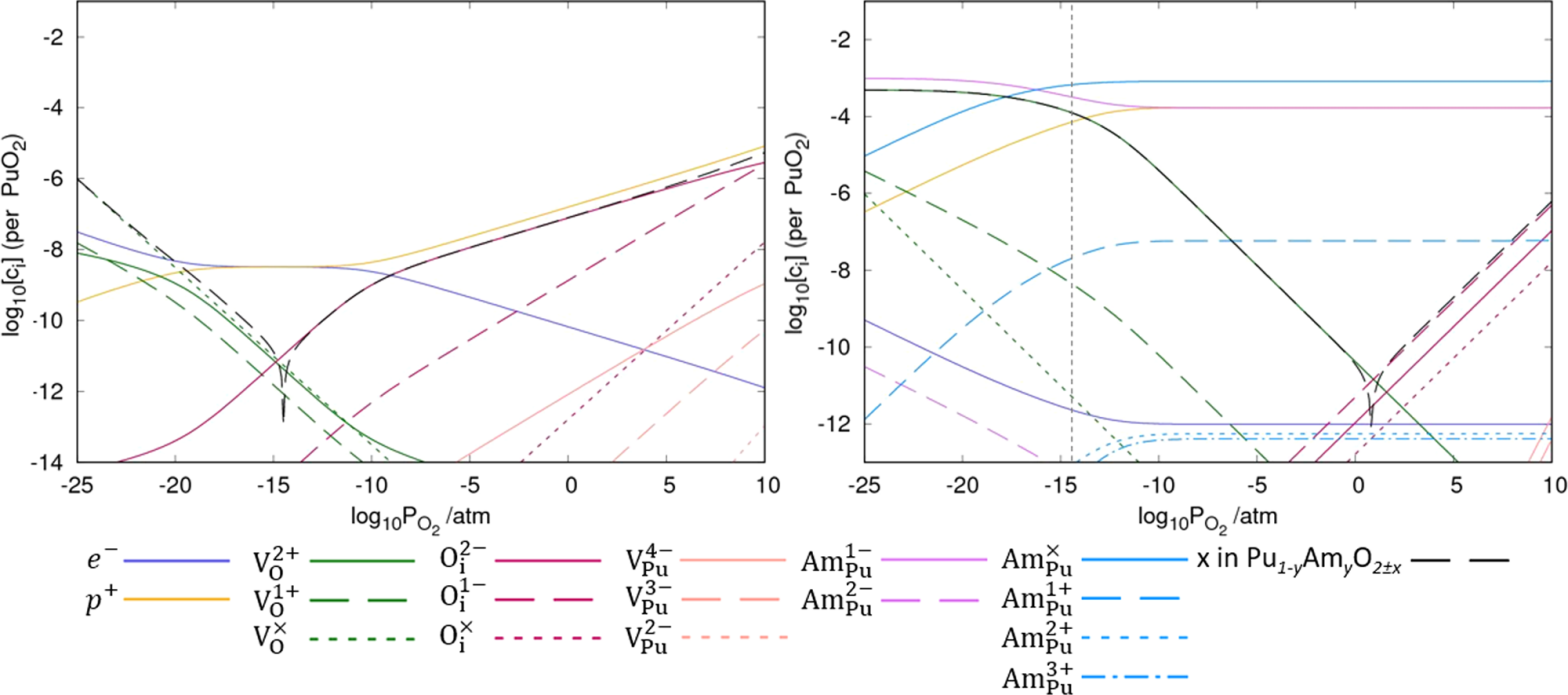
Brouwer diagrams showing the defect concentrations and value of *x* in (Pu_1–*y*_Am_*y*_)O_2±*x*_ as a function of oxygen partial pressure at a temperature of 1000 K and *y* value of 0.0 (left) and 0.001 (right). At partial pressures to the left of the vertical dashed line, (Pu,Am)O_2±*x*_ is predicted to be thermodynamically unstable with respect to Am_2_O_3_.

The Brouwer diagrams in Figure 3 show the defect concentrations in (Pu_1–*y*_Am_*y*_)O_2±*x*_ as a function of oxygen partial pressure at 1000 K with comparison made for *y* values of 0.0 and 0.001. At all values of oxygen partial pressure tested, Am is seen to be accommodated as substitutional defects on Pu sites, with concentrations of Am interstitials and O substitutions negligible to such an extent they are not shown on the Brouwer diagrams. The Brouwer diagram shows that as the O/M ratio in (Pu_1–*y*_Am_*y*_)O_2±*x*_ (*y* = 0.001) decreases, the dominant extrinsic defect transitions from Am_Pu_^×^ to Am_Pu_^1–^. As previously discussed, this transition corresponds to a reduction in the oxidation state for Am of +IV to +III. This supports the experimental work of Belin et al.^7^ as well as the model of Osaka et al.^5^ that find and predict all Am is reduced prior to Pu reduction when the O/M ratio decreases from stoichiometry. Charge compensation for the Am_Pu_^1–^ defect is provided by holes in the valence band at high oxygen partial pressures, before V_O_^2+^ defects compensate when their concentration becomes sufficiently high. Figure 3 shows that when Am (+IV) is the dominant oxidation state, the concentration of Am (III) ions remains elevated and stable: Am (III) contributes ∼17% to the total Am concentration in the region of stability in Figure 3. Consequently, the concentrations of holes also remain high to provide charge compensation, with concentrations several magnitudes higher than Am-free PuO_2_. It can therefore be said that Am behaves as a p-type dopant, acting to make PuO_2_ more conductive.

By comparing the two Brouwer diagrams in Figure 3, we observe that Am incorporation also impacts the intrinsic defect chemistry. The defect responsible for hypostoichiometry remains oxygen vacancies, however, the presence of Am is observed to alter the favored oxygen vacancy charge state; the Brouwer diagrams show that the doubly charged V_O_^2+^ defect dominates. In contrast, in PuO_2–*x*_, the neutral oxygen vacancy was preferred. Increased Am concentration promotes positively charged oxygen vacancies, as higher concentrations are required to charge-compensate (Am_Pu_^1–^). Hyperstoichiometry remains very low and accommodated by oxygen interstitials. In pure PuO_2+*x*_, the O_i_^2–^ interstitial is dominant. However, the dominant charge state is seen to be altered with the addition of Am: the O_i_^1–^ interstitial is now dominant. Prodan et al.^13^ have previously reported that O_i_^1–^ is the most energetically favorable charge state for the oxygen interstitial. Acting as a p-type dopant, increasing the Am concentration lowers the Fermi level of the system to such a degree that O_i_^1–^ becomes the interstitial with the lowest formation energy and V_O_^2+^ is the vacancy with the lowest formation energy.

**Figure 4:**
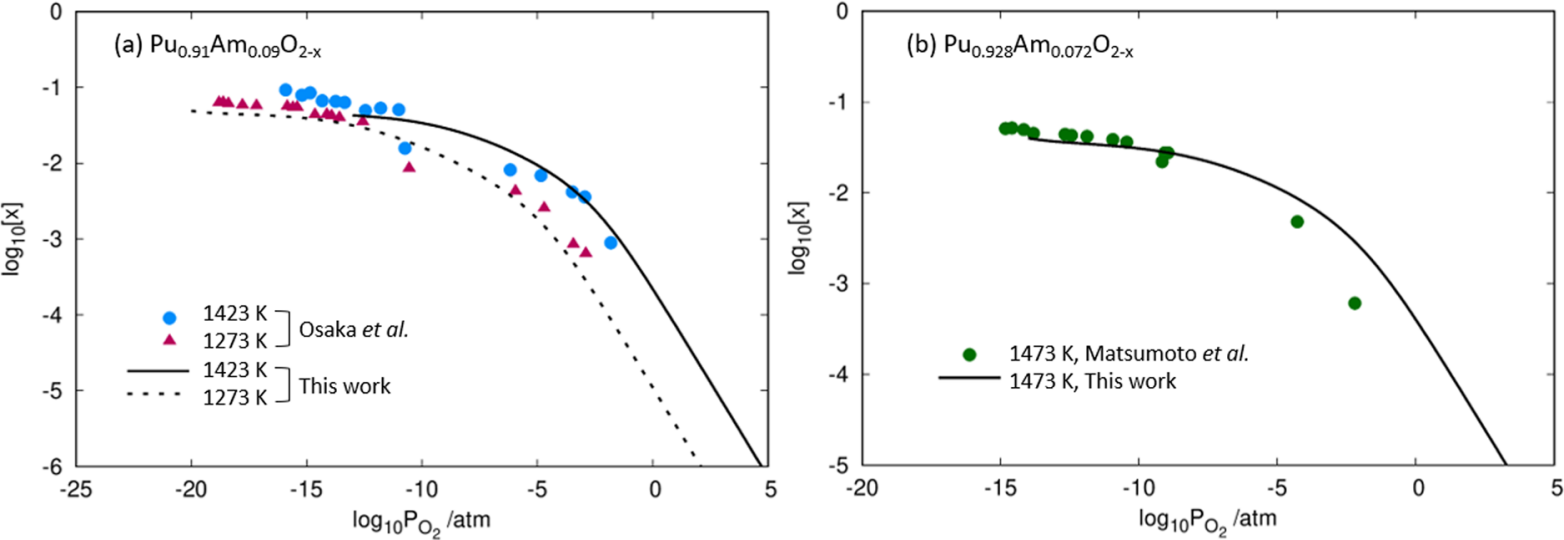
Values of *x* in (Pu_1–*y*_Am_*y*_)O_2–*x*_ as a function of oxygen partial pressure at *y* values of (a) 0.09 and (b) 0.072, with comparison to the experimental results of Osaka et al.^5^ and Matsumoto et al.^6^

To assess the reliability of the point defect model, the results are compared to the experimental studies of Osaka et al.^5^ and Matsumoto et al.^6^ who studied the O/M ratio in (Pu,Am)O_2–*x*_. By matching the temperature and Am concentrations of the experiments, it was possible to determine how *x* in (Pu,Am)O_2–*x*_ changes with oxygen partial pressure. The results are compared to these previous experimental works in Figure 4. It is seen that both the trends and absolute values of *x* are very well replicated by the model.

From Figures 3 and 4, we see an evolving dependence of [V_O_^2+^] and *x* on the oxygen partial pressure. At near-stoichiometry, our model shows that the presence of Am results in [e^–^] ≪ [h^+^], in contrast to Am-free PuO_2_, where [e^–^] = [h^+^] at near-stoichiometry. Therefore, to construct equations describing the defect chemistry of (Pu,Am)O_2–*x*_, we cannot say that [e^–^] = [h^+^] as suggested by Matsumoto et al.^6^ Instead, we propose that at low concentrations of V_O_^2+^ and near-stoichiometry, the formation of V_O_^2+^ is charge-compensated by the removal of holes, which exist at concentrations many orders of magnitude greater than V_O_^2+^ at near-stoichiometry. The defect reaction and the corresponding equilibrium constant (*k*_1_) are written as
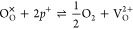
19
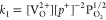
20We see in Figure 3 that at near-stoichiometry, the concentration of holes can be considered fixed. We can therefore show that [V_O_^2+^] (and *x* in (Pu,Am)O_2 – *x*_) is proportional to 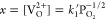
21Figure 3 shows that when oxygen partial pressures are low enough to cause sufficiently high concentrations of V_O_^2+^, reduction of Am begins. The defect reaction and corresponding equilibrium constant (*k*_2_) becomes
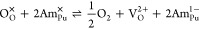
22
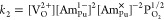
23In the reducing region, [Am_Pu_^1–^] = 2[V_O_^2+^]. Eq 23 can be rearranged to show that [V_O_^2+^] (and *x* in (Pu,Am)O_2 – *x*_) is proportional to both  and [Am_Pu_^×^]^2/3^ (see eq 24). This explains the curve we see in Figures 3 and 4: as Am (+IV) is reduced, the rate of V_O_^2+^ formation decreases and when reduction is complete, [V_O_^2+^] remains constant.
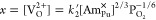
24As oxygen partial pressure is reduced further, the value of *x* in (Pu,Am)O_2–*x*_ will continue to evolve. The start of reduction in Pu is predicted to result in the formation of defect clusters;^6^ however, both cluster formation and the higher defect concentrations at low O/M ratios are beyond the capabilities of the point defect model.

**Figure 5:**
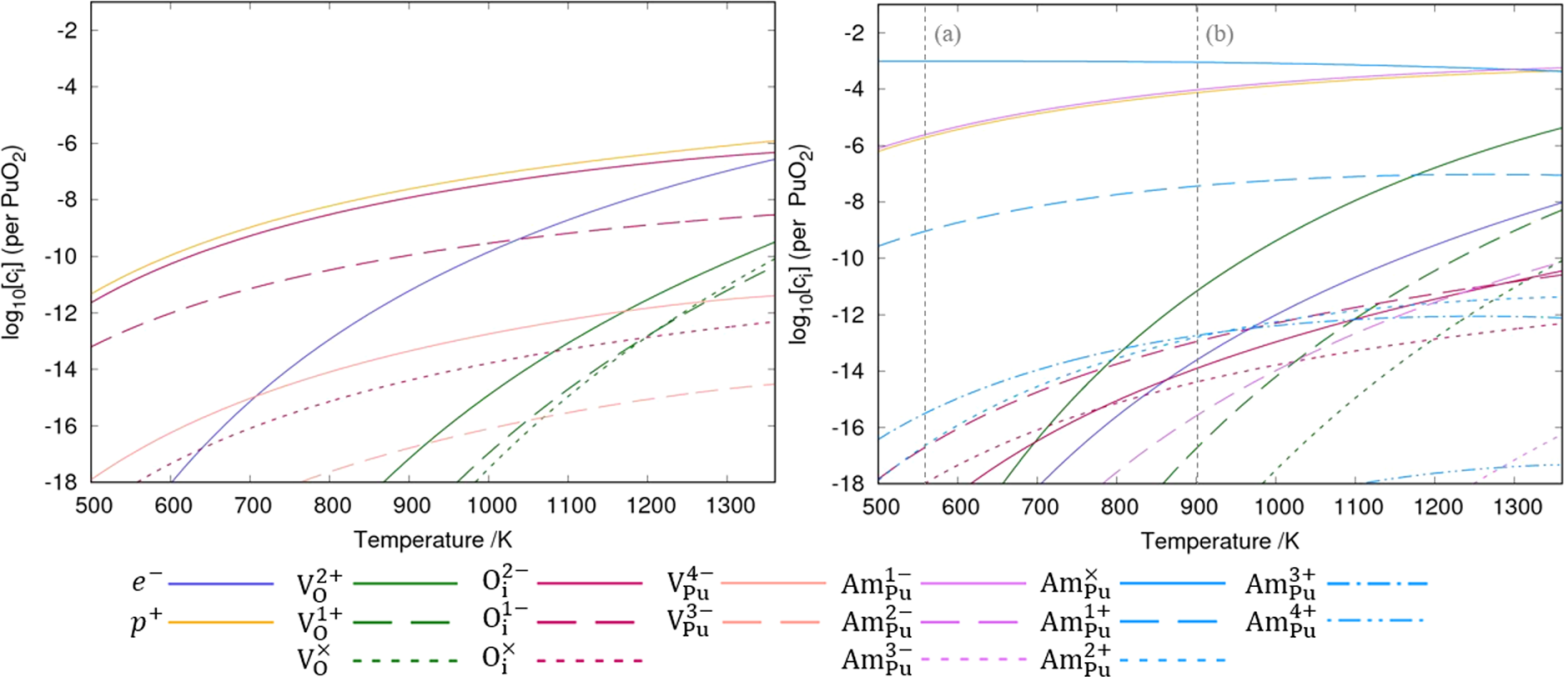
Defect concentrations in (Pu_1–*y*_Am_*y*_)O_2±*x*_ as a function of temperature at an oxygen partial pressure of 0.1 atm and *y* value of 0.0 (left) and 0.001 (right). At temperatures to the left of the vertical dashed lines (a) and (b), (Pu,Am)O_2±*x*_ is predicted to be thermodynamically unstable with respect to Am_2_O_3_ and AmO_2_, respectively.

Figure 5 shows the impact of varying temperature on the defect chemistry of (Pu_1–*y*_Am_*y*_)O_2±*x*_ where *y* = 0.0 or 0.001. Am (+III) concentration increases with temperature, becoming the dominant oxidation state at high temperatures. The Am (+IV)/Am (+III) ratio increases with decreasing temperature, and at low temperatures, the Am in (Pu_1–*y*_Am_*y*_)O_2±*x*_ is composed entirely of Am (+IV). This is supported by the recent finding of Emerson et al.^8^ who measured the Am L_3_-edge X-ray absorption near-edge structure (XANES) spectrum of aged PuO_2_ samples finding a spectrum characteristic of Am^4+^O_2_.

**Figure 6:**
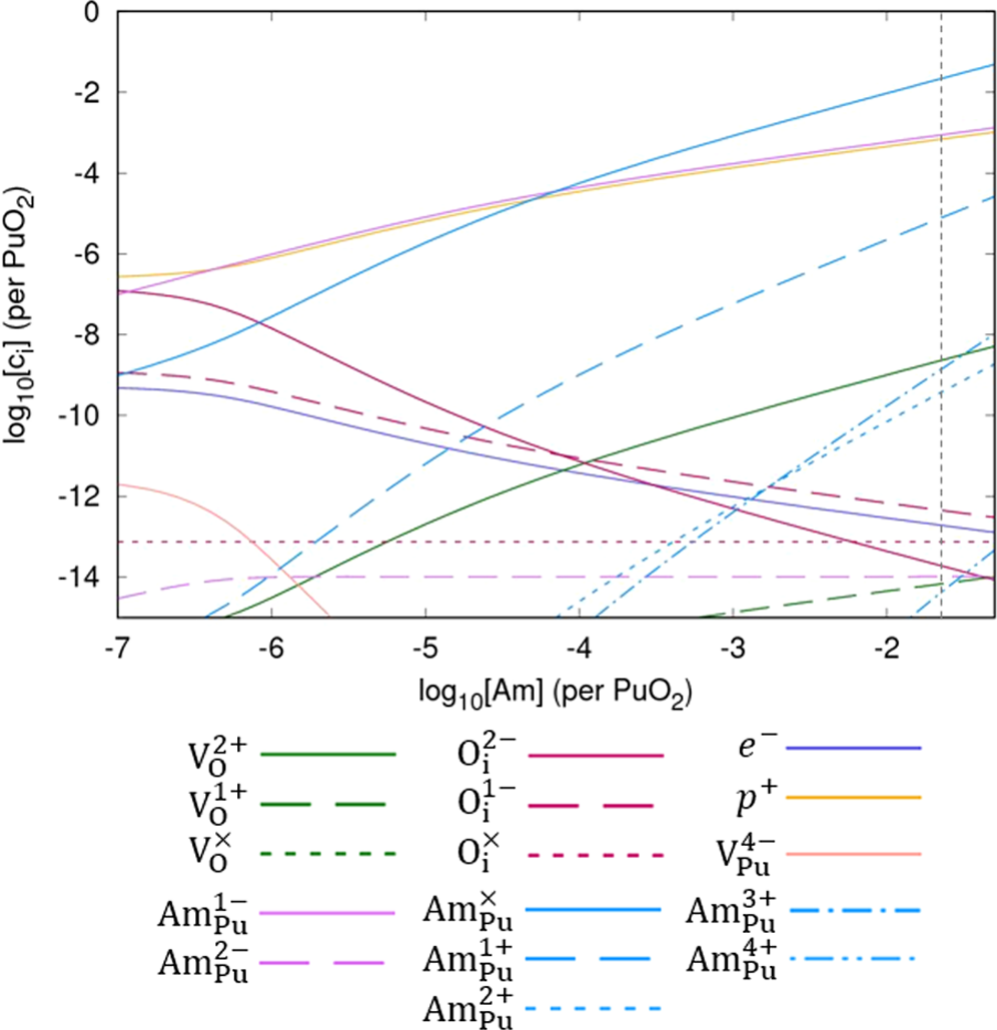
Defect concentrations in (Pu_1–*y*_Am_*y*_)O_2±*x*_ as a function of the concentration of Am at an oxygen partial pressure of 0.1 atm and temperature of 1000 K. At Am concentrations to the right of the vertical dashed line, (Pu,Am)O_2±*x*_ is predicted to be thermodynamically unstable with respect to AmO_2_.

The impact of varying the concentration of Am (*y* in (Pu_1–*y*_Am_*y*_)O_2±*x*_) is shown in Figure 6. Regardless of its concentration, Am is always accommodated in either the +IV or +III oxidation state, with the ratio of the two oxidation states varying quite significantly depending on the concentration of Am present. At very low concentrations, Am (+III) is the dominant oxidation state, whereas accumulation of Am in PuO_2_ results in the promotion of the +IV oxidation state. Although the Am (+IV) concentration increases more rapidly, the Am (+III) concentration also continues to increase as Am accumulates accompanied by a concomitant increase in conductivity of the material. Increasing Am concentration can be seen to create a more reducing environment; oxygen vacancy concentrations increase with increasing Am, and oxygen interstitial concentrations decrease. Therefore, under any condition, the O/M ratio is lower if Am concentration in PuO_2±*x*_ is increased. This is a similar result to that found in (U,Am)O_2±*x*_ where increased Am content is seen to hinder oxidation.^62^ PuO_2_ is much more resistant to oxidation than UO_2_, and we see here that adding Am further increases this resistance.

In Figures 3–6, the dashed vertical lines highlight the point at which the model predicts (Pu,Am)O_2±*x*_ is thermodynamically unstable and will decompose into a combination of two tested Am oxides: AmO_2_ and A-type Am_2_O_3_. The model predicts that at low oxygen partial pressures, low temperatures, or high Am concentrations, (Pu,Am)O_2±*x*_ becomes unstable. To precipitate out of the material, the Am oxides would require significant energy to overcome barriers to migration within (Pu,Am)O_2±*x*_. As it is found that at high temperatures (Pu,Am)O_2±*x*_ is stable, it is unlikely that under the conditions of instability predicted (Pu,Am)O_2±*x*_ would have the energy to decompose into the Am oxides, despite being thermodynamically favorable. Improvement may also be required in the DFT model for AmO_2_ and Am_2_O_3_. Specifically, the best approach to modeling with the DFT + *U* approach remains uncertain. Caution is therefore attached to the results regarding thermodynamic stability.

## Conclusions

The mode of Am incorporation within PuO_2_ and the impact Am makes to the defect chemistry of the host have been investigated using DFT and a point defect model. Under all conditions and Am concentrations investigated, Am is found to be accommodated on Pu vacancies, with Am existing in a combination of the (+IV) and (+III) oxidation states. Reduction in the O/M ratio of (Pu,Am)O_2±*x*_ is seen to change the dominant extrinsic defect from Am_Pu_^×^ to Am_Pu_^1–^ corresponding to the reduction of Am (+IV) to Am (+III). Am (+IV) is promoted by low temperatures and high Am concentrations. The addition of Am results in the concentration of holes in the valence band increasing by multiple orders of magnitude compared to Am-free PuO_2_, to provide charge compensation to Am (+III). It is, therefore, anticipated that the presence of Am increases the electrical activity of PuO_2_.

## Supporting Information Available

The evolving defect chemistry of (Pu,Am)O_2±*x*_: Appendix A: The impact of the *U* parameter in PBEsol + *U* on the density of states and DFT formation energies of defects in PuO_2_. Appendix B: Structural and electronic properties obtained in DFT simulation of Am oxides (PDF)
