# Supplementary material for: Evolving Defect Chemistry of (Pu,Am)O2±x
Source: J Phys Chem C Nanomater Interfaces. 2021 Jul 7;125(28):15560–8. doi: 10.1021/acs.jpcc.1c03274 (PMC8392350; doi:10.1021/acs.jpcc.1c03274)
Supplement: Supplementary file 1 — jp1c03274_si_001.pdf [file jp1c03274_si_001.pdf]

Supporting information for The Evolving Defect Chemistry of (Pu,Am)$O_{2\pm x}$.

William D. Neilson,† Helen Steele,‡ and Samuel T. Murphy*,†

†*Engineering Department, Lancaster University, Bailrigg, Lancaster, LA1 4YW, UK*

‡*Sellafield Ltd., Sellafield, Cumbria, CA20 1PG, UK*

E-mail: samuel.murphy@lancaster.ac.uk

Appendix A: The impact of the U parameter in PBEsol + U on the density of states and DFT formation energies of defects in PuO_2.

Figure 1 shows the density of states of PuO_2 calculated by the HSE06 hybrid functional and the PBEsol + U functional with varying values of U. Increasing U is seen to increase the band gap, but decrease the hybridisation of the O (p) and Pu(f) bands at the valence band maximum (VBM). The band gaps predicted for U values of 1 eV, 4 eV, and 7 eV are 0.52 eV, 2.23 eV, and 3.03 eV, respectively. The HSE06 functional predicts a band gap of 3.04 eV. PBEsol + U is unable to achieve a band gap exceeding 2.5 eV, as predicted by experiment[1] and hybrid DFT, whilst maintaining the hybridisation of the O (p) and Pu(f) bands at the VBM. We find that the DFT formation energy of a defect has a weak dependence on the selected U value and therefore the DOS. Table 1 demonstrates this, reporting the dependence on the DFT formation energy of the 2+ oxygen vacancy on the choice of U in the PBEsol + U functional.

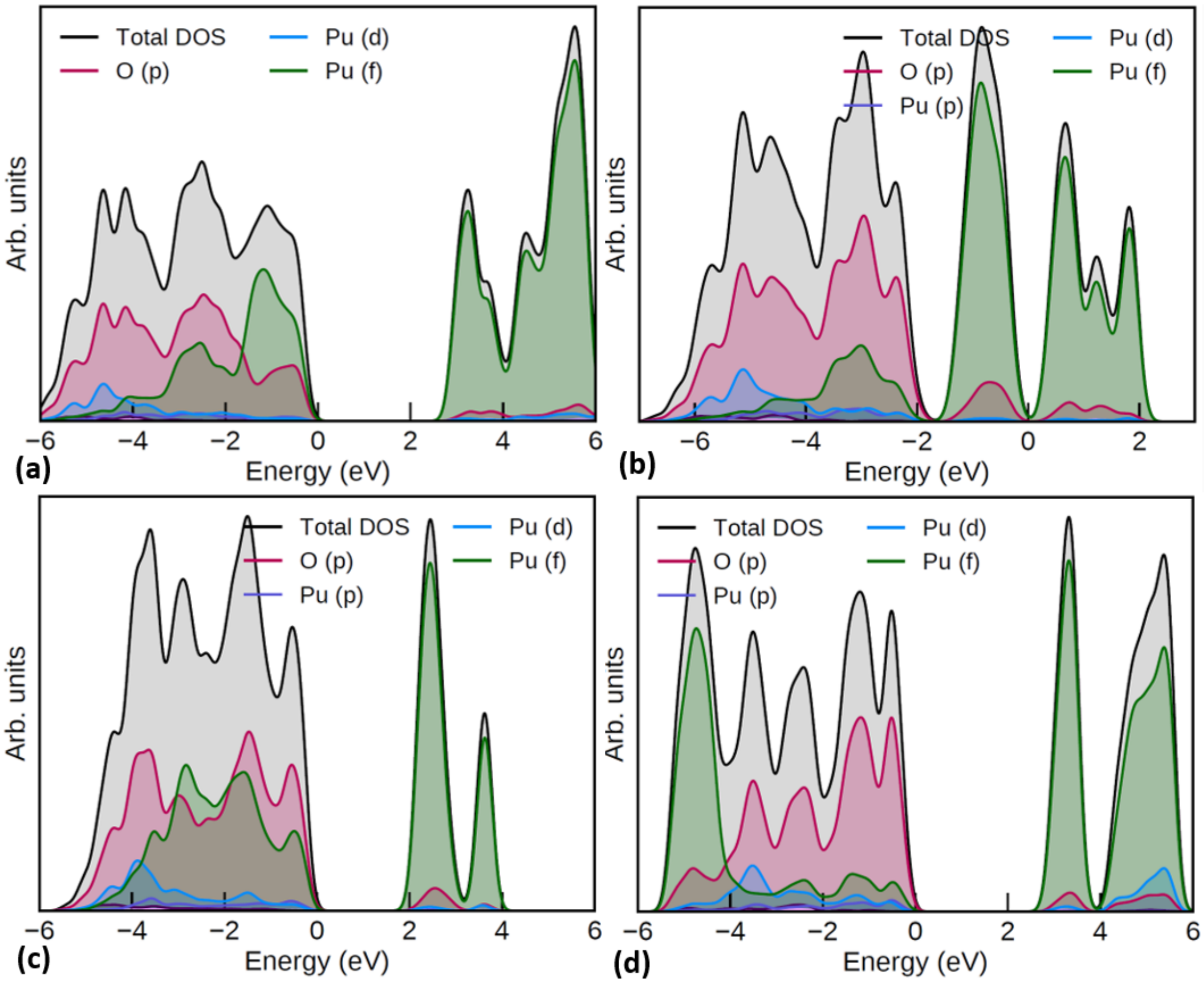

Figure 1: The density of states of PuO_2 calculated by (a) the HSE06 hybrid functional and the PBEsol + U functional where (b) $U = 1$ eV, (c) $U = 1$ eV, and (d) $U = 1$ eV.

Table 1: The impact of the choice of U in the PBEsol + U functional on the difference in energy between a defect-free and a 2+ oxygen vacancy -containing PuO_2 supercell.

U (eV)	DFT formation energy of V_O^{2+} defect (eV)	Difference in energy, compared to $U = 7$ eV (eV)	Percentage difference, compared to $U = 7$ eV
3	-9.68	0.15	1.54
4	-9.69	0.15	1.51
5	-9.71	0.12	1.27
6	-9.76	0.076	0.78
7	-9.84	0	0

Appendix B: Structural and electronic properties obtained in DFT simulation of Am oxides

Table 2: The lattice volume (Å^3), band gap (eV), magnetic moment (μ_B/Am ion) and space group for AmO_2 (transverse 3k AFM, SOI, $U = 4$ eV) and Am_2O_3 (longitudinal 1k AFM, SOI, $U = 4$ eV) calculated by PBEsol + U.

Oxide	Method	Lattice volume (Å^3)	Band gap (eV)	Magnetic moment (μ_B/Am ion)	Space group
AmO_2	PBEsol + U	155.25	1.30	4.85	$Pa\bar{3}$
	Experimental	155.29[2]	1.3[3]	-	$Fm\bar{3}m$[2]
Am_2O_3	PBEsol + U	73.66	2.57	5.66	$P\bar{3}m1$
	Experimental	74.73[4]	-	-	$P\bar{3}m1$[4]

References

(1) Mark McCleskey, T.; Bauer, E.; Jia, Q.; Burrell, A. K.; Scott, B. L.; Conradson, S. D.; Mueller, A.; Roy, L.; Wen, X.; Scuseria, G. E.; et al., Optical band gap of NpO_2 and PuO_2 from optical absorbance of epitaxial films. *J. Appl. Phys.* **2013**, *113*, 013515.

(2) Taylor, D. Thermal expansion data. *Trans. J. Br. Ceram. Soc.* **1984**, *83*, 32–37.

(3) Suzuki, C.; Nishi, T.; Nakada, M.; Akabori, M.; Hirata, M.; Kaji, Y. Core-hole effect on XANES and electronic structure of minor actinide dioxides with fluorite structure. *J. Phys. Chem. Solids* **2012**, *73*, 209–216.

(4) Chikalla, T. D.; Eyring, L. Phase relationships in the americium-oxygen system. *J. Inorg. Nucl. Chem.* **1968**, *30*, 133–145.
